# Molecular Design of FRET Probes Based on Domain Rearrangement of Protein Disulfide Isomerase for Monitoring Intracellular Redox Status

**DOI:** 10.3390/ijms241612865

**Published:** 2023-08-16

**Authors:** Maho Yagi-Utsumi, Haruko Miura, Christian Ganser, Hiroki Watanabe, Methanee Hiranyakorn, Tadashi Satoh, Takayuki Uchihashi, Koichi Kato, Kei-ichi Okazaki, Kazuhiro Aoki

**Affiliations:** 1 Exploratory Research Center on Life and Living Systems (ExCELLS), National Institutes of Natural Sciences, Okazaki 444-8787, Japan; 2 Institute for Molecular Science, National Institutes of Natural Sciences, Okazaki 444-8585, Japan; 3 The Graduate University for Advanced Studies, SOKENDAI, Okazaki 444-8787, Japan; 4 Graduate School of Pharmaceutical Sciences, Nagoya City University, Nagoya 465-8603, Japan; 5 National Institute for Basic Biology, National Institutes of Natural Sciences, Okazaki 444-8787, Japan; 6 Department of Physics, Nagoya University, Nagoya 464-8602, Japan; 7 Institute for Glyco-Core Research (iGCORE), Nagoya University, Nagoya 464-8601, Japan

**Keywords:** FRET-based biosensor, multidomain protein, protein disulfide isomerase, redox state

## Abstract

Multidomain proteins can exhibit sophisticated functions based on cooperative interactions and allosteric regulation through spatial rearrangements of the multiple domains. This study explored the potential of using multidomain proteins as a basis for Förster resonance energy transfer (FRET) biosensors, focusing on protein disulfide isomerase (PDI) as a representative example. PDI, a well-studied multidomain protein, undergoes redox-dependent conformational changes, enabling the exposure of a hydrophobic surface extending across the *b*’ and *a*’ domains that serves as the primary binding site for substrates. Taking advantage of the dynamic domain rearrangements of PDI, we developed FRET-based biosensors by fusing the *b*’ and *a*’ domains of thermophilic fungal PDI with fluorescent proteins as the FRET acceptor and donor, respectively. Both experimental and computational approaches were used to characterize FRET efficiency in different redox states. In vitro and in vivo evaluations demonstrated higher FRET efficiency of this biosensor in the oxidized form, reflecting the domain rearrangement and its responsiveness to intracellular redox environments. This novel approach of exploiting redox-dependent domain dynamics in multidomain proteins offers promising opportunities for designing innovative FRET-based biosensors with potential applications in studying cellular redox regulation and beyond.

## 1. Introduction

Most proteins in nature consist of two or more domains resulting from the duplication and combination of genes in the genome. Domain multiplicity enables sophisticated protein functions based on cooperative interactions and allosteric regulation, often coupled with environmental sensing for homeostatic control [1,2]. These functions are exerted through spatial rearrangements of the multiple domains. These properties of multidomain proteins could provide the basis for designing Förster resonance energy transfer (FRET) biosensors, as exemplified by Chameleon, an engineered protein based on calmodulin for sensing calcium levels [3].

A group of proteins, the protein disulfide isomerase (PDI) family, is one of the most extensively studied multidomain proteins [4,5]. Approximately 20 PDI family proteins have thus far been identified in humans, more than half of which contain more than one catalytically active or inactive thioredoxin-like domain. PDI, the representative protein of this family, consists of four thioredoxin-like domains, *a*, *b*, *b*’, and *a*’, and an acidic C-terminal extension [5,6,7]. The *a* and *a*’ domains contain a cysteine pair within a WCGHCK active-site motif involved in thiol/disulfide exchange reactions, while the *b* and *b*’ domains lack an active site motif. The accumulated experimental and computational data on PDI in humans or other organisms indicate that this protein undergoes redox-dependent conformational changes that are accompanied by the exposure of a hydrophobic surface that extends across the *b*’ and *a*’ domains and serves as the primary binding site for substrates [7,8,9,10,11,12,13,14,15,16,17,18,19]. The *b*’ and *a*’ domains are in contact in the reduced form of the *a*’ domain active site, shielding the hydrophobic surface, and their contact is weakened upon oxidation of the active site. This has prompted single-molecule FRET measurements for characterizing the redox-dependent conformational dynamics of PDI. In this approach, the full-length human PDI is site-specifically labeled with a pair of FRET dyes via click chemistry [20,21].

Redox reactions play a crucial role not only in cellular metabolism but also in intracellular processes such as transcription, translation, and signal transduction [22,23,24]. Therefore, using biosensors to detect changes in the redox state within living cells can contribute to understanding cell health, stress response, and potentially uncovering mechanisms underlying diseases. Herein, we developed an alternative approach to design FRET-based biosensors exploiting the redox-dependent conformational changes in PDI. In this approach, we used the *b*’ and *a*’ domains of thermophilic fungal PDI, as a minimal unit exhibiting redox-dependent domain rearrangement, and fused the domains to fluorescent proteins as a FRET acceptor and donor. This enabled in vivo expression of the FRET probes for monitoring intracellular redox environments. To optimize the design of the FRET-based biosensor, we perform experimental and computational characterization of the redox-dependent conformational dynamics of PDI-*b*’*a*’.

## 2. Results and Discussion

### 2.1. Dynamic Domain Rearrangement of PDI-b’a’

**Figure 1:**
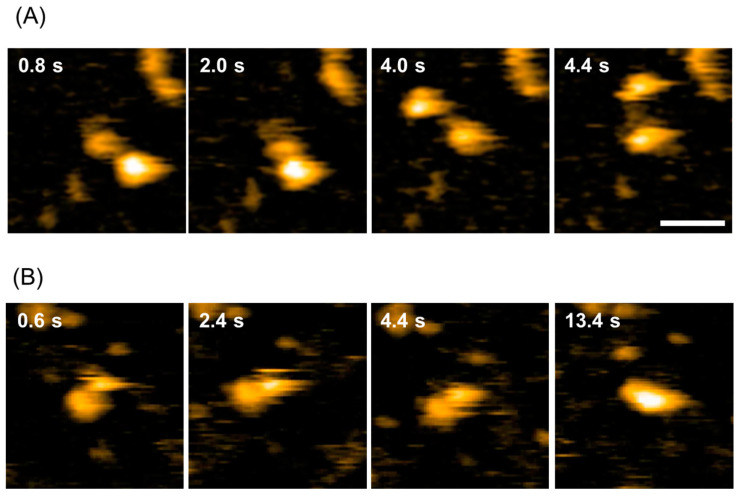
HS-AFM images of thermophilic fungal PDI-*b*’*a*’. Typical HS-AFM snapshots of (**A**) oxidized and (**B**) reduced forms of PDI-*b*’*a*’. Scale bar indicates 20 nm.

We first carried out real-time observations of redox-dependent conformational changes in thermophilic fungal PDI-*b*’*a*’ using high-speed atomic force microscopy (HS-AFM). Consistent with previous studies [11], the two domains underwent dynamic movements, changing positions with each other in the oxidized form (Figure 1A, Video S1A), whereas they stuck together and became one in the reduced form (Figure 1B, Video S1B). These results encouraged us to use PDI-*b*’*a*’ as a framework for designing FRET-based biosensors.

**Figure 2:**
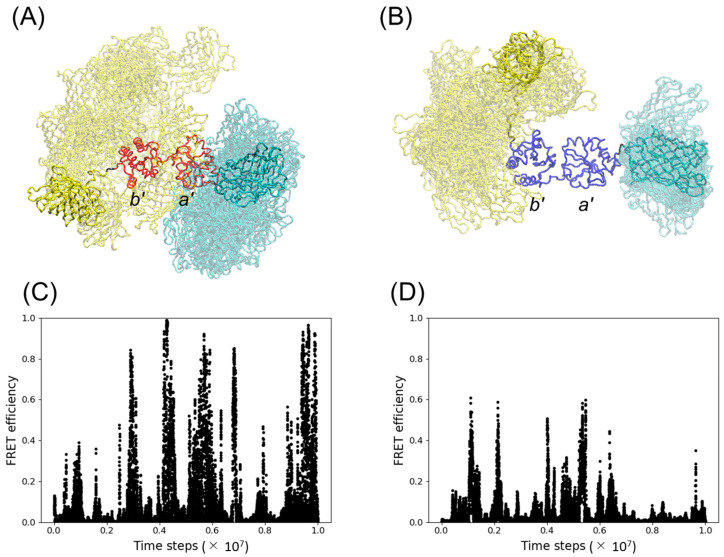
CG simulations of designed PDI probes. Conformational ensembles of YFP-PDI-*b*’*a*’-CFP are shown for the (**A**) oxidized and (**B**) reduced forms of thermophilic fungal PDI-*b*’*a*’. The YFP (yellow) and CFP (cyan) moieties in the conformational ensembles are shown after superimposing the *b*’ domain of PDI-*b*’*a*’. The FRET efficiencies from simulation snapshots are shown for the (**C**) oxidized and (**D**) reduced forms of YFP-PDI-*b*’*a*’-CFP. The proportion of calculated FRET efficiencies greater than 0.5 for the oxidized and reduced forms of YFP-PDI-*b*’*a*’-CFP was 5.1% and 0.2%, respectively.

To estimate the FRET efficiency in silico, we performed coarse-grained (CG) simulations of PDI-*b*’*a*’ fused to the fluorescent proteins CFP and YFP as a donor and acceptor, respectively. We attempted to employ the crystal structures of PDI-*b*’*a*’ as the initial structures for the simulation. In the crystal structures of thermophilic fungal PDI-*b*’*a*’, the crystallographically neighboring molecule hinders the potential domain–domain interaction, rendering the domain arrangement open irrespective of the redox state [8,15]. In contrast, the *b*’ and *a*’ domains in the crystal structure of the reduced form of human PDI (PDB ID: 4EKZ [13]) adopt a closed conformation consistent with the experimental and computational data in solution. Hence, we employed the crystal structure of the oxidized form of thermophilic fungal PDI-*b*’*a*’ (PDB ID: 3WT2 [8]) to construct an initial model of the oxidized form. We also conducted homology modeling based on the crystal structure of the reduced form of human PDI to construct an initial model of the reduced form of thermophilic fungal PDI-*b*’*a*’. Using these structures, we constructed initial structures of YFP-PDI-*b*’*a*’-CFP fusion proteins, where YPet, a YFP variant, and Turquoise-GL, a CFP variant, were fused to the N-terminus and C-terminus of thermophilic fungal PDI-*b*’*a*’, respectively. We performed CG simulations of the application of these models and estimated the FRET efficiency from the conformational ensembles of the oxidized and reduced forms. The results indicated that YFP and CFP are in various relative positions but often close together in the oxidized form, whereas they tend to be fixed at a distance in the reduced state, predicting that the FRET efficiency is significantly higher in the oxidized form (Figure 2). Inspired by these data, we carried out the recombinant expression of the YFP-PDI-*b*’*a*’-CFP fusion proteins to examine their potential utility as redox state-sensitive FRET probes.

### 2.2. In Vitro Characterization of PDI-b’a’-Based FRET Probes

**Figure 3:**
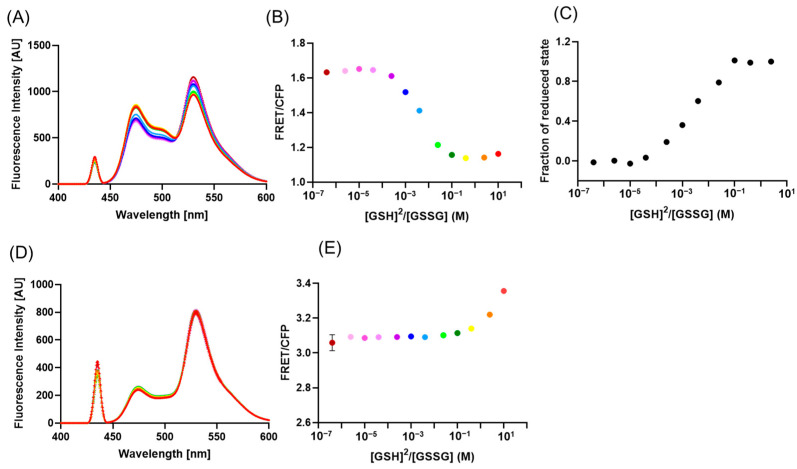
Fluorescence spectroscopic characterization of redox-titrated PDI-*b*’*a*’-based probes in vitro. (**A**) Fluorescence emission spectra (at 433 nm excitation) and (**B**) FRET efficiency of YFP-PDI-*b*’*a*’-CFP in the presence of varying ratios of GSH/GSSG. (**C**) The redox equilibria of PDI-*b*’*a*’ with different ratios of GSH/GSSG. (**D**) Fluorescence emission spectra (at 433 nm excitation) and (**E**) FRET efficiency of YFP-PDI-*b*’*a*’ ^C365S/C368S^-CFP in the presence of varying ratios of GSH/GSSG. The colors of the emission spectra in (**A**) and (**D**) correspond to the color of the point corresponding to each [GSH]^2^/[GSSG] concentration ratio in (**B**) and (**E**), respectively.

The recombinant YFP-PDI-*b*’*a*’-CFP proteins were expressed in *Escherichia coli* and subjected to in vitro evaluation of FRET efficiency under different redox conditions. The FRET efficiencies were measured by incubating purified recombinant YFP-PDI-*b*’*a*’-CFP proteins in redox buffers containing different ratios of [reduced glutathione, GSH] and [oxidized glutathione, GSSG] (Figure 3A,B). The redox equilibrium of thermophilic fungal PDI-*b*’*a*’ was also determined in a similar way (Figure 3C). As predicted with the CG simulations, the FRET efficiency of YFP-PDI-*b*’*a*’-CFP varied in response to the redox state of the *a*’ active site, being significantly higher under oxidized conditions when [GSH]^2^/[GSSG] was around 10^−6^. The alteration in FRET efficiency corresponding to the redox states closely related to the redox equilibrium of the *a*’ active site in PDI-*b*’*a*’. As a control, we generated a redox-insensitive mutant, YFP-PDI-*b*’*a*’^C365S/C368S^-CFP, in which the active site motif WCGHCK in the *a*’ domain was replaced with WSGHSK. HS-AFM revealed that PDI-*b*’*a*’ ^C365S/C368S^ adopted a rigid closed conformation similar to the reduced form of wild-type PDI-*b*’*a*’ (Video S2). As expected, the FRET efficiency of YFP-PDI-*b*’*a*’ ^C365S/C368S^-CFP was constant under varying redox conditions (Figure 3D,E). Unexpectedly, however, the FRET efficiency of this mutant was approximately 2–3 times higher than that of wild-type YFP-PDI-*b*’*a*’-CFP in the reduced state. This result suggests that the Cys-to-Ser mutation in the *a*’ active site affects its mode of interaction with the *b*’ domain, rendering the relative position or orientation of the flanking YFP and CFP preferable for FRET efficiency.

We also attempted to produce a series of circularly permutated YFP variants to optimize the relative dipole orientation of YFP and CFP. For this purpose, YPet in the FRET biosensors was replaced with three circularly permutated YPet variants—cp157YPet, cp173YPet, and cp229YPet—and these biosensors were successfully expressed in *Escherichia coli* and subjected to FRET measurements. All of these mutants showed a higher FRET efficiency under oxidizing conditions, suggesting that the redox-dependent domain arrangement of PDI-*b*’*a*’ was a significant factor in controlling FRET. Under both reducing and oxidizing conditions, the FRET efficiency was attenuated by the circular permutations (Figure S1). Therefore, we adopted the original YFP-PDI-*b*’*a*’-CFP using YPet as the FRET probe for in-cell applications.

### 2.3. In-Cell Characterization of PDI-b’a’-Based FRET Probes

**Figure 4:**
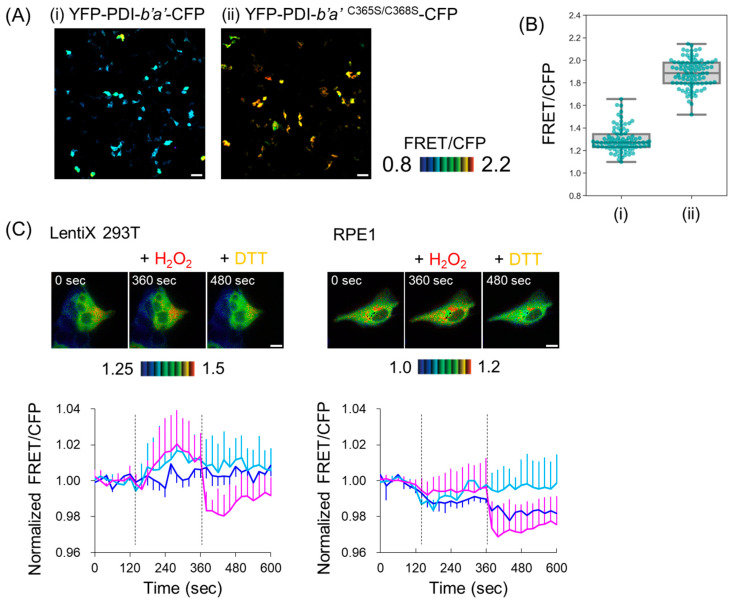
In-cell imaging experiments using a PDI-*b*’*a*’-based FRET probe. (**A**) The representative FRET/CFP ratio images of LentiX 293T cells expressing (**i**) YFP-PDI-*b*’*a*’-CFP (left) or (**ii**) YFP-PDI-*b*’*a*’ ^C365S/C368S^-CFP (right). Scale bar indicates 50 μm. Representative FRET/CFP ratio images are shown in the intensity-modulated display mode. (**B**) FRET efficiency of (**i**) YFP-PDI-*b*’*a*’-CFP and (**ii**)YFP-PDI-*b*’*a*’ *^C^*^365S/C368S^ -CFP. (**C**) LentiX 293T (left) and RPE1 (right) expressing YFP-PDI-*b*’*a*’-CFP were time-lapse-imaged and stimulated with 100 μM H_2_O_2_ after 120 s (time point: 120 s). Cells were subsequently treated with 1 mM DTT at 240 s following H_2_O_2_ stimulation (time point: 360 s). Representative FRET/CFP ratio images with YFP-PDI-*b*’*a*’-CFP are shown in the intensity-modulated display mode (upper). Scale bar indicates 10 μm. The FRET/CFP ratio of each cell was normalized by dividing it by the averaged FRET/CFP value before stimulation (lower). The mean and SD from 10 cells are plotted against time. Magenta, cyan, and blue lines show DTT treatment after H_2_O_2_ treatment, H_2_O treatment after H_2_O_2_ treatment, and H_2_O treatment after H_2_O treatment, respectively.

We characterized YFP-PDI-*b*’*a*’-CFP in mammalian cells to test its applicability as a FRET-based redox probe in vivo. We overexpressed YFP-PDI-*b*’*a*’-CFP or YFP-PDI-*b*’*a*’ ^C365S/C368S^-CFP in LentiX 293T cells and measured their FRET efficiency using fluorescence microscopy. While FRET was observed for both proteins, the efficiency was increased with the Cys-to-Ser mutation, as per the in vitro observations (Figure 4A,B). These data suggest that the microenvironmental changes in the *a*’ active site coupled with the spatial rearrangements of the *b*’ and *a*’ domains control the FRET efficiency in the intracellular environment.

We further examined the effects of redox perturbations on the intracellular FRET efficiency of YFP-PDI-*b*’*a*’-CFP. Specifically, LentiX 293T and hTERT-RPE1 (RPE1) cells expressing YFP-PDI-*b*’*a*’-CFP were observed in the presence and absence of hydrogen peroxide (H_2_O_2_), and dithiothreitol (DTT). FRET efficiency significantly increased with H_2_O_2_ and decreased with DTT in Lenti-X 293T cells, whereas RPE1 cells did not show an increase in efficiency with H_2_O_2_ treatment (Figure 4C). These results suggested a difference in redox states or their sensitivity to oxidative stress between cell lines. Other possibilities causing the change in FRET efficiency should be carefully considered. For example, the accumulation of cellular metabolite, including free radical particles or reactive oxygen species, might have influenced the FRET efficiency of YFP-PDI-*b*’*a*’-CFP, or a pH change might have caused protonation of the chromophore in YPet, such that YPet did not fluoresce. In any case, future studies will be needed to clarify what was responsible for the change in FRET efficiency.

Because of the importance of monitoring intracellular redox status in basic cell biology, various redox-sensing probes have been developed based on engineered fluorescent proteins for live-cell imaging [25]. A series of fluorescent protein variants with an artificially introduced cysteine pair (such as roGFP, rxYFP, and Oba-Q) have been created to probe the redox status of a cell based on ratiometric imaging [26,27,28]. For the sensitive detection of intracellular redox changes against the background, single-molecule FRET-based approaches have been developed using a pair of fluorescent proteins connected via a redox-sensing region derived from the chloroplastic regulatory protein CP12 or the yeast transcription factor Yap1, which undergoes redox-dependent conformational change [29,30]. In either case, the disulfide formation in the redox-sensing region brings two fluorescent proteins into close spatial proximity, thereby enhancing FRET efficiency. In contrast, the FRET biosensors developed in this study are based on redox-dependent domain rearrangement and exhibit higher FRET efficiency in oxidized form, complementing previously developed FRET probes.

### 2.4. Concluding Remarks

In multidomain proteins, local perturbations such as ligand binding and site-directed mutation cause gross conformational changes accompanied by domain rearrangements, providing spectroscopic probes for monitoring intracellular environments as well as in vitro applications including drug screening [3,20,21,31,32,33]. Experimental and computational characterization of the conformational dynamics of multiple domains associated with fluorescence proteins is required to design effective biosensors. In this study, we employed CG simulations, which successfully predicted the redox-dependent change in FRET efficiency of the PDI-*b*’*a*’-based probe. This approach can be applied to designing new FRET biosensors based on other PDI family protein members that exhibit distinct redox potentials. Moreover, the redox potential of their active sites can be modulated via mutagenesis. However, it is generally difficult to predict the possible impacts of active site modification on domain–domain interactions, as exemplified by the effect of the Cys-to-Ser mutation in the *a*’ active site. Further liaison between experimentation and theory will be necessary to explore new vistas in the design of biosensing probes based on multidomain proteins.

## 3. Materials and Methods

### 3.1. Plasmids

The cDNA encoding PDI-*b*’*a*’ (residues 208–449) from *Humicola insolens* was amplified using PCR (KOD One PCR Master mix, TOYOBO), and subcloned into the pCR4Blunt-Topo vector (Zero Blunt™ TOPO™ PCR Cloning Kit, Invitrogen). The inserted cDNA was confirmed with sequencing and further subcloned in between the YFP and CFP of the pCAGGS-YFP-MCS-CFP vector [34] with the conventional ligation method (Ligation High ver.2, TOYOBO). The cDNAs of YFP include YPet [35] and its circularly permutated variants cp157YPet, cp173YPet, and cp229YPet, which were produced based on the circularly permutated variants of Venus [36]. The cDNA of CFP was derived from Turquoise-GL [37].

### 3.2. Bacterial Expression and Purification of Recombinant Proteins

The expression and purification of the PDI-*b*’*a*’ (residues 208–449) and PDI-*b*’*a*’ ^C365S/C368S^ from *Humicola insolens* were performed as previously described [8,11]. YFP-PDI-*b*’*a*’-CFP and their variants were expressed with an N-terminal hexahistidine tag using the pCold-I vector in *E. coli* and purified using a Ni^2+^-immobilized affinity column (cOmplete^TM^ His-Tag Purification Resin, Roche) from the soluble lysate. After the cleavage of the hexahistidine tag with TEV protease, these proteins were purified using a HiLoad Superdex 200 column (Cytiva) in 50 mM Tris–HCl (pH 8.0).

To oxidize the *a*’ active site, the purified protein (1 mg/mL) was dialyzed using 50 mM Tris-HCl (pH 8.0) containing 0.1 mM oxidized glutathione for 10 days. To reduce the *a*’ active site, the protein was dissolved in a buffer containing 2–4 mM DTT (Sigma Aldrich, St. Louis, MO, USA).

### 3.3. HS-AFM

HS-AFM images of PDI-*b*’*a*’ and PDI-*b*’*a*’ ^C365S/C368S^ were acquired in tapping mode using a laboratory-built HS-AFM apparatus [38] and a short cantilever (Olympus: BL-AC7; 6–7 μm long, 2 μm wide, and 90 nm thick) at room temperature. The oxidized and reduced forms of PDI-*b*’*a*’ were observed at a concentration of 14 nM in 50 mM Tris–HCl buffer (pH 8.0) without and with 1 mM DTT, respectively. PDI-*b*’*a*’ ^C365S/C368S^ was observed at a concentration of 12 nM in 50 mM Tris–HCl buffer (pH 8.0) without and with 1 mM DTT. The sample droplet was placed on a freshly cleaved mica or mica treated with 0.01–0.025% 3-aminopropyltriethoxysilane (APTES-mica) and left to adsorb for 3 min. Before imaging, the substrate was washed with 20 µL of 50 mM Tris-HCl buffer to remove any unbound species.

### 3.4. Fluorescence Measurement In Vitro

Fluorescence emission spectra were recorded with 7 µM YFP-PDI-*b*’*a*’-CFP (or its variant) in 50 mM Tris–HCl buffer (pH 8.0) at 25 °C using a fluorescence spectrometer (Hitachi F2700, Japan) in the presence and absence of 4 mM DTT or a varying concentration ratio of GSH/GSSG. Emission spectra were recorded at 400–600 nm with an excitation wavelength of 433 nm. FRET peaks were observed at 530 nm with an excitation wavelength of 433 nm. FRET efficiency was calculated using FRET efficiency = fluorescent intensity of FRET peak (emission wavelength of 530 nm)/fluorescent intensity of donor (CFP, emission wavelength of 475 nm).

### 3.5. Cell Culture

LentiX 293T and hTERT RPE1 cells were purchased from the Takara Bio (Kusatsu, Japan) and American Tissue Culture Collection (ATCC) (Manassas, VA, USA), respectively, and were maintained in Dulbecco’s Modified Eagle’s Medium (DMEM), high glucose (FUJIFILM Wako Pure Chemical Corporation, Osaka, Japan; NACALAI TESQUE, INC., Kyoto, Japan) supplemented with 10% fetal bovine serum (Sigma Aldrich).

LentiX 293T and RPE1 cells were seeded on 35 mm glass-bottom dishes (Asahi Techno Glass, Shizuoka, Japan) or four-compartment 35 mm glass-bottom dishes (Greiner Bio-One, Kremsmünster, Austria). After seeding, the plasmids were transfected using 293fectin (Invitrogen, Waltham, MA, USA) according to the manufacturer’s instructions. Then, the cells were cultured for at least 24 h, and the culture medium was replaced with FluoroBrite DMEM (Life Technologies, Carlsbad, CA, USA) supplemented with 1x GlutaMax (Life Technologies) and 0.2% fetal bovine serum for approximately 3 h.

### 3.6. FRET Imaging

For short-term imaging experiments, images were acquired with an IX81 inverted microscope (Olympus, Tokyo, Japan), which was equipped with a Retiga 4000R cooled Mono CCD camera (QImaging, Surrey, Canada), a Spectra-X light engine illumination system (Lumencor, Inc., Beaverton, OR, USA), an IX2-ZDC laser-based autofocusing system (Olympus), a UPlanSApo 60x/1.35 oil objective lens (Olympus), a MAC5000 controller for filter wheels and XY stage (Ludl Electronic Products, Hawthorne, OR, USA), an incubation chamber (Tokai Hit, Fujinomiya, Japan), and a GM-4000 CO_2_ supplier (Tokai Hit). The following filters and dichroic mirrors were used: for CFP, an FF01-438/24 excitation filter (Semrock, Lake Forest, IL, USA), an XF2040 445DRLP dichroic mirror (Omega, Biel, Switzerland), and an FF01-483/32-25 emission filter (Semrock); for FRET, an FF01-438/24 excitation filter (Semrock), an XF2040 445DRLP dichroic mirror (Omega), and an FF01-542/27 emission filter (Semrock). The microscopes were controlled using MetaMorph software ver. 7.8.11.0 (Molecular Devices, San Jose, CA, USA). The images were acquired every 20 s for 10 min. An amount of 100 uM H_2_O_2_ (Wako) or H_2_O was added at 2 min, followed by the addition of 1 mM DTT or H_2_O at 6 min.

### 3.7. CG Simulations

The initial structures for the fused protein YFP-PDI-*b*’*a*’-CFP were prepared as follows: The oxidized form of PDI-*b*’*a*’ was obtained from thermophilic fungal PDI-*b*’*a*’ structures (PDB ID: 3WT2 [8]). The reduced form of PDI-*b*’*a*’ was constructed using homology modeling with MODELLER using the reduced form of the human PDI structure (PDB ID: 4EKZ [13]) as well as the *a*’ and *b*’ domains in 3WT1 [8].

The YFP/CFP structure was modeled from the structure of Venus (PDB ID: 1MYW [39]). Seven missing residues at the C-terminal of YFP (EGMNELY), two residues between YFP and PDI-*b*’ (LE), four residues between PDI-’*a*’ and CFP (AGGR), and other missing residues were modeled with MODELLER [40].

We performed CG molecular dynamics simulations of YFP-PDI-*b*’*a*’-CFP using the CafeMol program, in which each amino acid is coarse-grained into one bead and structure-based potentials are employed [41]. The structure-based potentials were fine-tuned based on information from atomic AMBER energy and the structural database. In this study, we used the latest AICG2+ energy function [42]. Langevin dynamics at 300 K was used for the time integration of 10^7^ steps.

For the calculation of FRET efficiency from simulation snapshots, we used the side-chain orientation of the chromophore Tyr/Trp in YFP/CFP that was approximated by a vector from the Tyr/Trp Cα atom to the Ser147 Cα atom position to represent the transition dipole. From the distance and orientation factors of the YFP and CFP dipoles, the FRET efficiency was calculated using the Förster equation:

(1) $$E_{eff}=\frac{1}{1+r^{6}/\left({R_{0}}^{6}\alpha \right)}$$

where r is the distance of the dipoles; $R_{0}$ is the Förster distance with random orientation set to 53 Å; and $\alpha =\frac{\kappa^{2}}{\left(2/3\right)}$, the relative orientation factor from the random orientation.

## Data Availability

The data presented in this study are available on request from the corresponding author.

## Supplementary Materials

The supporting information can be downloaded at: https://www.mdpi.com/article/10.3390/ijms241612865/s1.
