# Supplementary material for: Molecular Design of FRET Probes Based on Domain Rearrangement of Protein Disulfide Isomerase for Monitoring Intracellular Redox Status
Source: Int J Mol Sci. 2023 Aug 16;24(16):12865. doi: 10.3390/ijms241612865 (PMC10454184; doi:10.3390/ijms241612865)
Supplement: Supplementary file 1 [file ijms-24-12865-s001.zip › ijms-2560048-supplementary.pdf]

Supplementary Materials

(A)

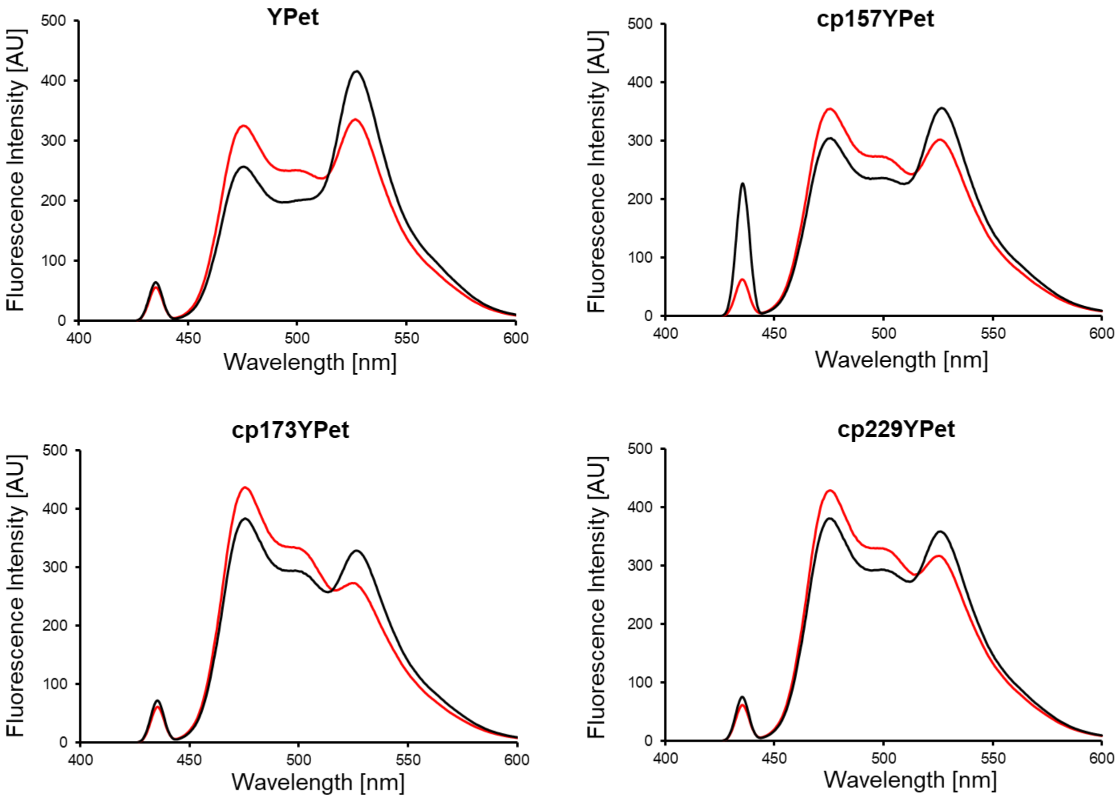

(B)

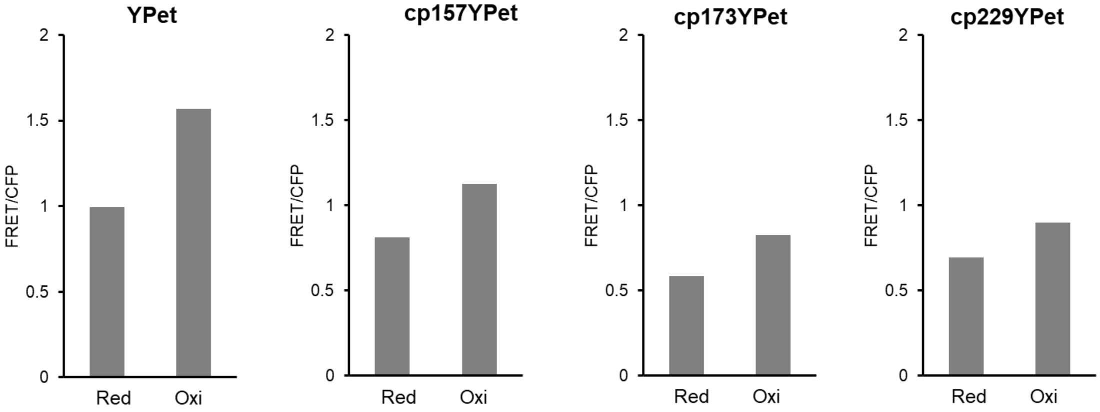

Fig S1.

(A) Fluorescence emission spectra (at 433 nm excitation) of YFP-PDI-*b'a'*-CFP circularly permutated variants in which the YFP part is wild-type YPet, cp157YPet, cp173YPet, and cp229YPet in the presence (red) and absence of DTT (black). (B) FRET efficiency of the reduced and oxidized states of YFP-PDI-*b'a'*-CFP variants.

Video S1 HS-AFM videos of the (A) oxidized and (B) reduced forms of thermophilic fungal PDI-*b'a'*.

Video S2 HS-AFM videos of the thermophilic fungal PDI-*b'a'* $^{C365S/C368S}$ in the (A) absence and (B) presence of DTT.
